# Wifebeating

**Published:** 1912-12

**Authors:** 


Wifebeating. Wm. F. Waugh of Chicago, *Wis. Med. Rec.*, Oct., 1912, puts up a plausible plea for this treatment of Mrs. Booze, Mrs. Giddy and Mrs. Frowsy.

